# Does a simplified algorithm and integrated HCV care model improve linkage to care, retention, and cure among people who inject drugs? A pragmatic quality improvement randomized controlled trial protocol

**DOI:** 10.1186/s12879-024-08982-1

**Published:** 2024-01-19

**Authors:** Stacey L. Klaman, Job G. Godino, Adam Northrup, Sydney V. Lewis, Aaron Tam, Carolina Carrillo, Robert Lewis, Eva Matthews, Blanca Mendez, Letty Reyes, Sarah Rojas, Christian Ramers

**Affiliations:** Laura Rodriguez Research Institute - Family Health Centers of San Diego, 1750 Fifth Avenue, San Deigo, CA 92101 USA

**Keywords:** Hepatitis C, Hepatitis C treatment, People who inject drugs, Mobile medical clinic, People experiencing homelessness, Treatment initiation, Linkage to care, Quality improvement

## Abstract

**Background:**

As many as 2.4 million Americans are affected by chronic Hepatitis C Virus (HCV) in the United States.In 2018, the estimated number of adults with a history of HCV infection in San Diego County was 55,354 (95% CI: 25,411–93,329). This corresponded to a seroprevalence of 2.1% (95% CI: 2.1–3.4%). One-third of infections were among PWID. Published research has demonstrated that direct-acting antivirals (DAAs) have high efficacy and can now be used by primary care providers to treat HCV. In addition, limited evidence exists to support the effectiveness of simplified algorithms in clinical trial and real-world settings. Even with expanded access to HCV treatment in primary care settings, there are still groups, especially people who inject drugs (PWID) and people experiencing homelessness, who experience treatment disparities due to access and treatment barriers. The current study extends the simplified algorithm with a streetside ‘one-stop-shop’ approach with integrated care (including the offer of buprenorphine prescriptions and abscess care) using a mobile clinic situated adjacent to a syringe service program serving many homeless populations. Rates of HCV treatment initiation and retention will be compared between patients offered HCV care in a mobile clinic adjacent to a syringe services program (SSP) and homeless encampment versus those who are linked to a community clinic’s current practice of usual care, which includes comprehensive patient navigation.

**Methods:**

A quasi-experimental, prospective, interventional, comparative effectiveness trial with allocation of approximately 200 patients who inject drugs and have chronic HCV to the "simplified care" pathway (intervention group) or the "usual care" pathway (control group). Block randomization will be performed with a 1:1 randomization.

**Discussion:**

Previous research has demonstrated acceptable outcomes for patients treated using simplified algorithms for DAAs and point-of-care testing in mobile medical clinics; however, there are opportunities to explore how these new, innovative systems of care impact treatment initiation rates or other HCV care cascade outcomes among PWID.

**Trial registration:**

We have registered our study with ClinicalTrials.gov, a resource of the United States National Library of Medicine. This database contains research studies from United States and other countries around the world. Our study has not been previously published. The ClinicalTrials.gov registration identifier is NCT04741750.

## Background

With as many as 2.4 million Americans affected by chronic Hepatitis C Virus (HCV) [1], it is one of the most common blood-borne diseases in the United States [1]. There are numerous studies assessing cascades of care for dried blood spot and point of care testing in different settings including in prisons, community and mobile units [2, 3]. Reports of new infections have increased for the seventh consecutive year, with 44,300 new HCV infections in 2017—a 7.5% increase from 2016 and a 375% increase since 2010 [4, 5]. Nationwide, rates of new infections have risen dramatically among young adults in their 20s and 30s [4], the same cohorts most affected by the opioid epidemic, with as many as 70% of new infections related to injection drug use [5]. Local/regional rates of new infection mirror these national surveillance findings. In 2018, the estimated number of adults with a history of HCV infection in San Diego County was 55,354 (95% CI: 25,411–93,329). This corresponded to a seroprevalence of 2.1% (95% CI: 2.1–3.4%). One-third of infections were among PWID.” [6]. Expanded prevalence estimates indicate a population prevalence rate for HCV of 2.0% to 2.7% in San Diego County, suggesting that approximately 65,000 to 88,000 individuals in the region are likely HCV-infected, with these infections concentrated in the PWID population, with an estimated 65% seroprevalence [6].

These alarming increases in new HCV infections and very high seroprevalence in PWID demand effective treatment delivered to populations that have historically been difficult to reach and, due to a constellation of barriers to care, are characterized by disparities in HCV screening, linkage to care, and treatment access. Advances in HCV treatment and care (e.g., the development of highly effective direct-acting antivirals—DAAs) show promise for treating these populations and have led to worldwide HCV elimination goals [7], as well as local/regional elimination campaigns—with Family Health Centers of San Diego’s (FHCSD) Hepatitis treatment team providing elimination leadership through the county-wide End Hep C San Diego Initiative. While the American Association for the Study of Liver Diseases/Infectious Diseases Society of America (AASLD/IDSA)’s guidelines have long been considered the gold standard for Hepatitis C (HCV) diagnosis, workup, and treatment (and remain appropriate for HCV specialists, especially in complex cases of HCV infection) recent World Health Organization (WHO) HCV guidelines [8] and published consensus statements [9] call for implementation of streamlined and simplified algorithms for HCV care, delivered in an integrated primary care setting. They highlight that most patients, particularly younger people who inject drugs (PWID), have a low risk of cirrhosis and do not require genotyping if treated with pangenotypic regimens. Furthermore, emerging evidence indicates that PWID have been able to achieve high cure rates relative to their high transmission risk. Therefore, it is critical to make concerted efforts to target these patient populations to achieve HCV elimination [10–12]. While these findings offer a hopeful outlook for treatment, many of these patients may be lost to follow-up due to overly complex, time-intensive, and costly evaluations [10].

While research indicates that DAAs have high efficacy and safety and can now be used by primary care providers to treat HCV [13–15], limited evidence exists to support the safety and effectiveness of simplified algorithms, nor patient uptake and efficiency of the approach in a real-world setting. For example, even with expanded access to HCV treatment in primary care settings, there are still groups, especially PWID and people experiencing homelessness, who experience treatment disparities due to access and treatment barriers [16]. In particular, those who are pre-contemplative with respect to medications for opioid use disorder, actively injecting drugs and street homeless, transportation needs and competing priorities serve as major barriers to accessing testing and treatment for HCV. In response to this need, new and innovative methods to simplify the care cascade and expand screening and treatment access are being introduced, including use of mobile medical clinics.

At least one study has shown that point of care testing in a mobile medical clinic resulted in significantly higher rates of HCV infected patients being linked to HCV treatment in 30 days compared to standard phlebotomy HCV testing [17]. While simplified HCV diagnostic and treatment algorithms have shown promise in improving treatment initiation and completion rates [9–11, 18, 19], studies have largely been confined to the traditional clinic environment. The current study combines the simplified treatment algorithm with streetside treatment as the point of service of our syringe service program (SSP) thus exploring a novel, patient-centered approach. Therefore, the proposed study will test the effectiveness of a simplified HCV algorithm with integrated care (including the offer of buprenorphine prescriptions and abscess care), in a street-based mobile clinic setting among PWID and people experiencing homelessness, in increasing treatment initiation and retention rates. Rates of HCV treatment initiation and retention will be compared between patients managed using a simplified algorithm of HCV care in a mobile clinic versus those who are linked to FHCSD’s community clinic current practice of usual care, which includes comprehensive patient navigation.

The primary objective of this Hepatitis C pragmatic quality improvement comparative effectiveness trial is to determine the effectiveness of using a simplified HCV algorithm coupled with integrated care for PWID, delivered in a mobile medical clinic, to increase the number of patients who initiate treatment compared to a concurrent control group receiving usual care.

The secondary objectives are to determine if the simplified algorithm and mobile treatment delivery results in improvement of: (1) HCV therapy completion; (2) SVR12 rates; (3) initiation rates for Medication Assisted Treatment (MAT) for opioid use disorder; (4) persistence on MAT; and (5) receipt of abscess care. Additional exploratory aims are to determine the average time to linkage to care and HCV treatment initiation (and other HCV care cascade outcomes) and compare these times to a similar historical sample. Data to be generated from this study includes the number of patients with chronic HCV who are linked to care, start DAA therapy, complete therapy, return for SVR12, and attain SVR12. Data also will be collected on general engagement in care, initiation of MAT, and abscess care. Results will provide a fuller understanding of the impact of innovative elimination strategies through simplification of the HCV patient care cascade. Results also will have policy implications for payer regulations concerning the safe, effective, and efficient treatment of HCV, as well as clinical implications for innovative ways to engage marginalized patients.

## Methods/design

**Fig. 1:**
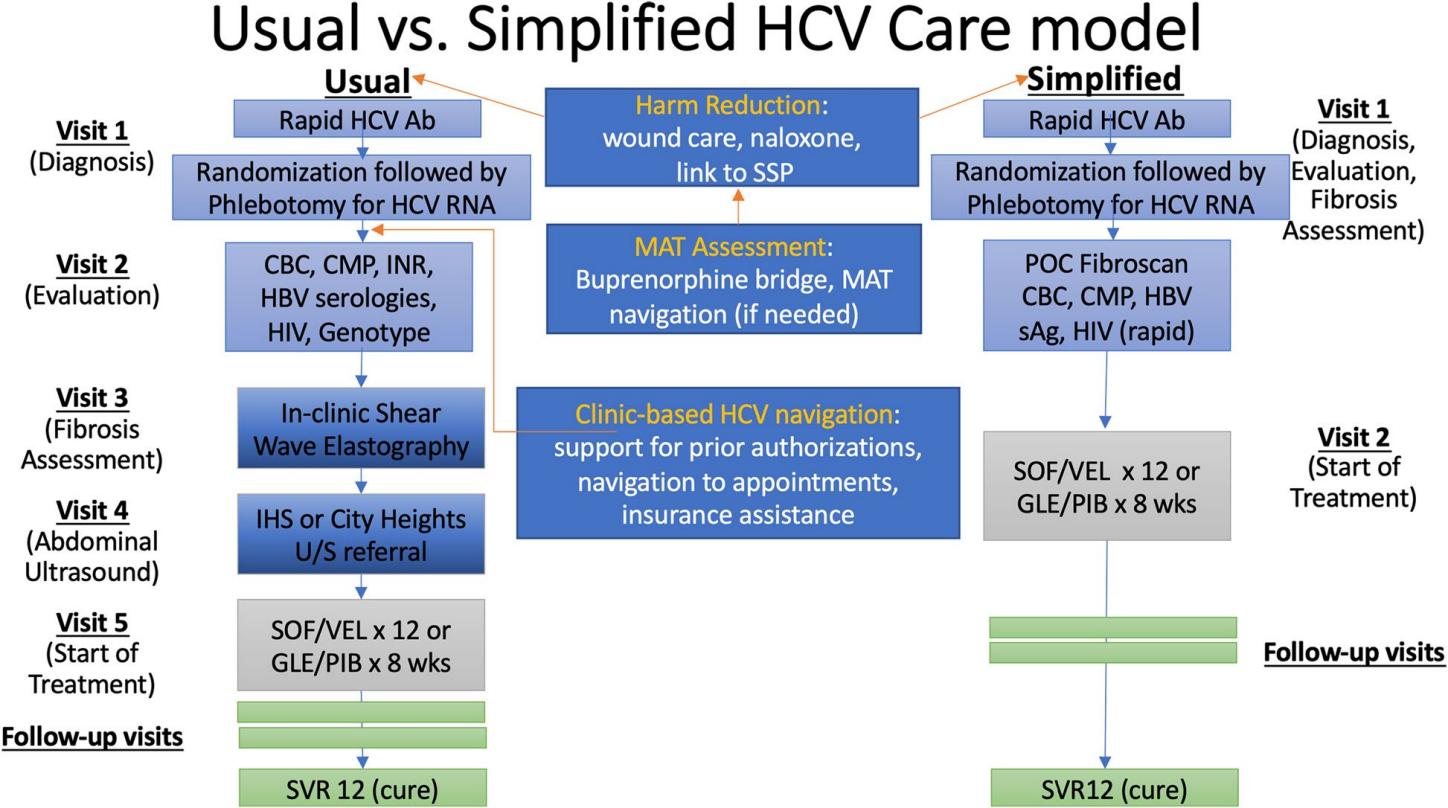
Usual Care vs. Simplified Care

This study is a quasi-experimental, prospective, interventional, comparative effectiveness trial with random allocation of approximately 200 patients who inject drugs and test positive for HCV Ab to one of two groups. One group receives the “simplified care” pathway (intervention group), while the other group of patients receives the “usual care” pathway (control group). The design of the trial and flow of patients are shown in Fig. 1.

### Sample size justification

To ensure that the proposed study has adequate power to determine the effectiveness of the simplified algorithm to determine the effectiveness of the simplified algorithm to improve HCV treatment initiation, i.e., the percentage of patients who are HCV RNA positivie and initiate treatment, the sample size was calculated based on a two-sided, two-sample proportion test with 80% power at a significance level of 5%. According to existing FHCSD data, between 20-25% of HCV infected patients (HCV RNA positive) initiate care [20]. Because the proposed treatment strategy addresses both barriers and facilitators of HCV treatment initiation, we believe that a greater than 15-20% improvement in care initiation is achievable. Thus, when the proportion of patients who initiate treatment in the control group is 20%, the target sample of approximately 200 patients (100 per study group) will provide sufficient power to detect a difference of approximately 17.5%. Furthermore, existing FHCSD data indicates that in the syringe exchange setting, approximately 15-20% of patients who take a rapid finger stick test are HCV Ab positive and of those, roughly 75% are HCV RNA positive [21]. Therefore, we will aim to administer approximately 1,350 tests during patient recruitment in order to identify approximately 200 patients who are HCV RNA positive and will be offered treatment.

### Recruitment

Patients are recruited from FHCSD's mobile SSP population (N >2,369). Data from a survey of SSP clients shows that 94% of the population is low income, 70% are homeless or unstably housed, and 78% have co-occurring mental health and/or substance use disorders. 90% are uninsured or covered by Medi-Cal. Patients are potentially eligible to participate in the study and excluded if they are age <18 years, and have one or more of the following: undetectable HCV RNA, a life expectancy of < 6 months, known clinical or laboratory characteristics indicating decompensated cirrhosis, known hepatocellular carcinoma. Additionally, they are excluded if they are pregnant.

Patients are recruited and enrolled in the study once or twice a week (Tuesdays and or Thursdays) for 30 months. We expect to stop enrollment in September 2023.

### Randomization

In order to ensure equal allocation across the two trial groups and reduce the likelihood of contamination bias, we will use a permuted blocked randomization 25] procedure to determine which patients seeking care will receive the “simplified care” pathway at the mobile clinic and which patients will receive the "usual care" pathway in a clinic. A statistician without knowledge of patient characteristics created a computer-generated list comprised of permuted blocks of 4, 8, or 12 that contain half of each of the two trial groups per block in a random order. The randomization list was incorporated into a computer program that members of the study coordination team use for enrollment and automated randomization of patients. Allocation is concealed from the study coordination team, researchers, and patients until the interventions are assigned. It is not possible to blind patients to which intervention they receive. However, researchers assessing the baseline characteristics of patients and the primary outcome of the trial remain blinded to group assignment.

### Intervention

The interventions consist of 12 weeks of HCV treatment and 12 weeks of follow-up. The experimental group (“simplified care,” *n* = 100) will consist of patients who will be offered a simplified HCV workup, Medication Assisted Treatment (MAT), and abscess care to meet patients’ urgent and survival needs while capitalizing on the ability to initiate HCV therapy when they are most motivated to engage in care. A complete HCV workup will be offered immediately upon a preliminary positive HCV Ab test at a mobile medical clinic parked adjacent to the mobile SSP van, or at a second homeless tent site located several blocks from the SSP van. On-site workup will include: CBC, CMP, INR, HCV RNA, HBV serologies, a point of care HIV test, and point of care liver fibrosis measurement (Fibroscan mini). HCV genotype also will be drawn if required by the patient’s insurance prior authorization requirements.

Patients will be assessed for opioid use disorder and, if appropriate, will be offered 7-day starter packs of buprenorphine/naloxone and linkage into FHCSD’s MAT program. Concurrently, patients will receive skin infection and abscess care if needed. Upon completion of the 20-minute visit, patients will have undergone a workup sufficient to initiate a prior authorization request for DAAs from their payer and an appointment for full-scope MAT follow-up in an FHCSD clinic if indicated. Rapid HCV blood tests, patient coordination, authorization efforts with insurance companies, and scheduling of appointments, follow-up, and ancillary support services will be conducted by the HCV Patient Navigator who will provide continuity of patient care which is expected to foster high rates of patient linkage to, and retention in, HCV care. Confirmatory HCV tests and other needed blood tests will be performed by the Certified Phlebotomy Technician. Blood samples will be transported to FHCSD’s centralized laboratory for processing.

Clinical assessment and bedside Fibroscan will be conducted by a medical provider with experience and training in HCV treatment, Fibroscan assessment, and MAT, including an active DEA X-waiver, in order to prescribe buprenorphine if indicated. A complete abdominal ultrasound will be recommended only for patients determined to have advanced fibrosis (F3/F4) by Fibroscan. Patients found to have decompensated cirrhosis, as evident from clinical history of encephalopathy, coagulopathy, gastrointestinal bleeding, ascites, or by laboratory criteria (Child Turcotte-Pugh score) will be referred to an FHCSD clinic site for further evaluation, formal hepatology consultation, and treatment outside of the study.

Within two to three weeks, insurance authorization will have been obtained and patients will be able to start HCV treatment on a subsequent visit to the mobile clinic. The HCV Patient Navigator will extract baseline and confirmatory test results from the electronic medical record and record them in a study database, disclose confirmatory test results to the patient, conduct post-test counseling, provide educational materials, address barriers to attending medical appointments, and schedule follow-up medical visits at the mobile medical clinic to complete a course of HCV therapy.

After treatment initiation, patients must return to be seen every 2-4 weeks at the treating clinician’s discretion to receive their DAAs and for monitoring and adherence support as needed. Additional lab draws will not be required but will be at the discretion of the treating provider and will align with currently accepted best practices. Patients with cirrhosis, renal impairment, or hepatitis B co-infection will have lab draws at a minimum of every 4 weeks. Throughout treatment, FHCSD’s Clinical Management Information System (CMIS) will trigger reminders to contact patients for appointments and the HCV Patient Navigator will contact patients and continuously address barriers to treatment, as needed. In the case of missed appointments, the HCV Patient Navigator will reach out to patients to reschedule appointments and address barriers. Treatment duration will be either 8 or 12 weeks, depending on the specific regimen. HCV treatment regimens will be selected according to current AASLD/IDSA guidelines (www.hcvguidelines.org) and insurance preference. Twelve weeks after completion of HCV therapy, an SVR12 HCV RNA and SVR12 CMP tests will be obtained.

The concurrent control group (“usual care,” *n* = 100) will receive treatment consistent with FHCSD’s current practice. Patients with a positive HCV Ab rapid finger stick test will have immediate phlebotomy performed for HCV RNA. Results will be disclosed the following week upon a second visit and HCV RNA positive patients will be offered referrals and assisted with comprehensive patient navigation to attend an initial HCV evaluation at an HCV-treating FHCSD clinic. At patients’ first visit after linkage to an FHCSD clinic, baseline CBC, CMP, chronic Hepatitis panel, HCV Genotype (if required), Hepatitis A Ab, and INR tests will be performed. Another visit will be required to conduct Fibroscan/SWE, and another separate visit will be required to perform abdominal ultrasound. When a full workup is completed, the prior authorization request will occur as per usual FHCSD clinic-based process. As in the intervention group, HCV treatment regimens will be selected according to current ASSLD/IDSA guidelines (www.hcvguidelines.org) and insurance preference. When DAA medications are approved and delivered (typically a month after the preliminary positive HCV Ab rapid finger stick test), patients can begin treatment. Patients will be seen at week #2, 4, 8, and 12 as per usual FHCSD practice. In the fourth treatment week, HCV RNA and CMP tests will be conducted. At the end of treatment (either 8th or 12th treatment week), end-of-treatment HCV RNA and CMP tests will be conducted. Twelve weeks after treatment is completed, an SVR12 HCV RNA and CMP tests will be conducted. As in the experimental group, an HCV Patient Navigator will conduct patient coordination, assist with prior authorization efforts with insurance companies, schedule follow-up appointments, and attempt to alleviate other barriers. The HCV Patient Navigator will extract test results from the electronic medical record and enter them into a study database; disclose confirmatory test results to the patient; conduct post-test counseling; provide educational materials, address barriers to attending medical appointments, and schedule medical visits. Throughout treatment, CMIS will trigger reminders to contact patients for appointments and the HCV Patient Navigator will contact patients and address barriers to treatment, as needed. In the case of missed appointments, the HCV Patient Navigator will reach out to patients to reschedule appointments and address barriers. Patients in need of buprenorphrine/naloxone, MAT, skin infection treatment, and abscess care will have access to these services as is current practice within FHCSD’s fixed-site clinic site.

### Measures

Measures recorded at baseline only will include age, sex, ethnicity, race, marital status, employment status, military history, education, housing status, household income, tobacco and alcohol use, and medical history will be measured through self-report and entered into the EHR.

The primary outcome is HCV treatment initiation, defined as initiating treatment with a Direct-Acting Antiviral (DAA) during 6 months of follow-up.

Secondary outcomes include: (1) HCV therapy completion, defined as completing a full treatment course of 8 or 12 weeks of DAA therapy within a period of 16 weeks after treatment initiation. As this is a real-world study, HCV treatment is assumed with medication dispensation and patient report; no specific measures such as pill counts, therapeutic drug level monitoring, or MEMS caps were used in the context of this study; (2) SVR12 rates, defined as an undetectable HCV RNA on or after the SVR12 time point (at least 12 weeks following DAA completion); (3) initiation rates for MAT for Opioid Use Disorder among those eligible for MAT, defined as attendance of at least one visit to an FHCSD clinic for treatment of Opioid Use Disorder during the first 3 months of follow-up; and (4) persistence on MAT, defined as > 3 visits for MAT during 6 months of follow-up, and (5) receipt of abscess care, among those with abscesses, during 6 months of follow-up. Additional measures to be generated from the EHR include the time to linkage care, start of DAA therapy, and completion of DAA therapy; (6) general engagement in care defined as the number of visits for any reason during the study period.

Serious adverse events also will be assessed and defined as any hospitalization, emergency room visit, and/or death.

### Statistical analysis

Statistical analyses will be conducted using the latest version of the statistical software platform R and/or STATA and will be based on the intention-to-treat principle [22, 23]. All tests of significance will be two-sided and a *p*-value of 0.05 will be considered statistically significant. Summary statistics (e.g., mean, standard deviations, proportions) will be calculated for all variables of interest. Outliers will be assessed and variables whose distributions depart significantly from normality will be transformed. Appropriate non-parametric alternatives will be considered if parametric assumptions fail. No planned interim analyses for efficacy or futility will be conducted in this study.

Comparison of the proportion of patients initiating HCV treatment between study groups will be presented as a risk difference with 95% confidence intervals [21, 24]. Adjusted risk differences and 95% confidence intervals will be computed using a Poisson model with robust standard errors. The comparison of time to initiating HCV treatment between study groups will be assessed using time-to-event methods, including Kaplan–Meier survival curves and Cox proportional-hazards models [25, 26]. Similar statistical procedures will be used to assess differences between study groups in secondary outcomes. Additionally, we will conduct exploratory analyses to examine factors that may moderate the effectiveness of the simplified algorithm on study outcomes. Moderators (e.g., age, sex, housing status, chronic disease status) will illuminate for whom and under what conditions the simplified algorithm may have been effective. Adding interaction terms to the logit models assessing the adjusted risk differences will test moderation.

### Benefits

PWID in both arms of this study who might not normally have access to HCV testing and treatment, or to abscess care and MAT, will be given the opportunity to receive such care. At the community level, this may result in a reduction of transmission of HCV to others and increased likelihood of the eradication of HCV.

### Risks

Potential risks will not derive from any research activities. The probability and magnitude of harm or discomfort anticipated in the research are not greater in and of themselves than those ordinarily encountered in daily life or during the performance of routine care. Thus, by definition, the risks are no more than minimal.

The risk of loss of confidentiality is no greater than that experienced by any FHCSD patient. The confidentiality of clinician and patient data is a top priority for the research team. FHCSD has substantial training requirements for all physicians and staff that include strict adherence to HIPAA. All members of the research team will be trained to ensure confidentiality and adherence to standardized procedures. All research staff directly involved with the collection and storage of research materials will complete CITI training and internal FHCSD HIPAA and data security training. In addition, the research study will comply with policies established by FHCSD’s Data Security Plans. Any information obtained about clinicians and patients during this study will be treated as strictly confidential to the full extent permitted by applicable law and in accordance with HIPAA regulations. Only trained members of the research team will have access to patient identifiers and data collected.

No data other than that which is collected within routine care will be used in this study. To ensure confidentiality, a research code number will be assigned to each patient and information that may identify them. The research code numbers will only be provided to qualified study investigators. Files linking names and other identifying information to data will be electronically saved using technology that prevents unauthorized individuals from accessing and understanding it. Patients' identifying information will not be included in any data sets used for analyses. This will ensure that patients are not easily connected to the study results.

If a patient’s information is printed, it will be kept locked and accessible only to certified research staff. When study results are published no personally identifying information (according to HIPAA guidelines) will be revealed.

## Discussion

Previous research has demonstrated an optimistic view of outcomes for patients treated with DAAs [8] and the use of point-of-care testing in mobile medical clinics [15]; however, there are opportunities to explore how these new, innovative systems of care impact treatment initiation rates or other HCV care cascade outcomes among PWID, a population that frequently experiences disparities in access to treatment [14]. This quality improvement RCT, which has the potential to be replicated by other FQHCs, will be one of the first to examine the potential effects of a simplified algorithm and integrated HCV care model on the linkage to care, retention, and cure among PWID.

## Data Availability

The datasets used and/or analysed during the current study are available from the corresponding author on reasonable request.

## Abbreviations

AASLD/IDSA: American Association for Study of Liver Diseases and the Infectious Diseases Society of America
CBC: Complete Blood Count
CMP: Comprehensive Metabolic Panel
DAA: Direct-Acting Antiviral
DEA: Drug Enforcement Agency
EHR: Electronic Health Record
FHCSD: Family Health Centers of San Diego
FQHC: Federally Qualified Health Center
GLE/PIB: Glecaprevir/Pibrentasvir
HIPAA: Health Insurance Portability and Accountability Act
HBV: Hepatitis B Virus
HCV: Hepatitis C Virus
HIV: Human Immunodeficiency Virus
HIS: Health Information System
INR: International Normalized Ratio
MAT: Medication Assisted Treatment
POC: Point of Care
PWID: People Who Inject Drugs
RCT: Randomized Controlled Trial
RNA: Ribonucleic Acid
SOF/VEL: Sofosbuvir/Velpatasvir
SSP: Syringe Services Program
SVR: Sustained Viral Response
SWE: Shear Wave Elastography
WHO: World Health Organization
